# Perampanel during pregnancy: Description of four cases

**DOI:** 10.1016/j.ebr.2021.100490

**Published:** 2021-10-08

**Authors:** A.M. Alicino, G. Falcicchio, G. Boero, G. Santarcangelo, T. Francavilla, M. Trojano, A. La Neve

**Affiliations:** a Department of Basic Medical Sciences, Neurosciences and Sense Organs, University of Bari, Bari, Italy; b Complex Structure of Neurology, SS Annunziata Hospital, Taranto, Italy; c Territorial Neurology Service, Policoro, ASM Matera, Italy

**Keywords:** Epilepsy, Perampanel, Pregnancy, Anti-seizure medications (ASMs), Neonatal major malformations

## Abstract

Perampanel (PER) is approved in Italy as an adjunctive treatment for focal-onset seizures (FOS) and generalized tonic-clonic seizures (GTCs), and it could be an alternative to valproate in young women diagnosed with idiopathic generalized epilepsy. Nevertheless, clinical data about the outcome of pregnancies in women exposed to PER are lacking. Here, we report retrospectively collected data from four women suffering from FOS who were exposed to PER during pregnancy. Three pregnancies were carried out with PER as add-on therapy during the entire gestation (8 mg/day in two patients and 6 mg/day in one), without seizure frequency variations. The fourth patient started PER 2 mg/day as monotherapy during the 13th week of pregnancy due to seizure relapse and continued it until delivery with complete seizure control. All pregnancies showed good outcomes, and their newborns did not possess major congenital malformations. Apgar scores and auxological parameters at birth were normal. Fetal pathology in follow-up during pregnancies was absent in all cases. In our patients PER was well tolerated and appeared safe for the fetuses and did not result in major malformations or adverse events at birth. Nevertheless, this is a report involving a small number of patients and it does not suggest the general use of PER is safe during pregnancy.

## Highlights

• Data about only 96 cases of pregnancies exposed to PER are available to date.
• Few information are available about perinatal metrics.
• We report perinatal metrics and newborn vital signs of 4 pregnancies exposed to PER.

## 1 Introduction

The use of newer anti-seizure medications (ASMs) during pregnancies is growing worldwide [1] due to their increasing utilization in epilepsy and other diseases, such as bipolar disorders and neuropathic pain [2].

Therefore, there is a need to assess their safety *in utero* to evaluate the balance among their therapeutic effects and the potential risk of adverse events in the exposed foetus.

Perampanel (PER) is a second-generation ASM licensed in Italy in 2012 as adjunctive therapy for focal-onset seizures (FOS) and generalized tonic-clonic seizures (GTCs). Interestingly, due to numerous studies on its efficacy in generalized epilepsies [3], PER represents a potential alternative to valproate, where the high risk for a teratogenic effect has been demonstrated [4]. Preclinical studies in pregnant rats and rabbits indicate that PER may cause post-implantation loss, diverticulum of the intestine, and delays in physical development [5].

There have not been any adequate clinical studies to assess the outcomes of pregnancies in women exposed to PER during gestation; only single reports or data about few cases of pregnancies are available, none of which are from the European Registry of Antiepileptic Drugs and Pregnancy (EURAP) [6].

While waiting for future data from large systematic collections of pregnancies with PER, single clinical experiences can be very useful to guide therapy.

We report data from four pregnancies of women affected by epilepsy who were exposed to PER.

## 2 Material and methods

We retrospectively reviewed clinical records and identified four pregnancy exposures to PER. Three patients were followed by the Epilepsy Centre of the University of Bari, and one patient was followed by the Neurological Outpatient Ambulatory of Policoro Hospital. We evaluated seizure frequency before and during gestation, maternal and fetal side effects, screening tests during pregnancy, pregnancy course, delivery, birth outcomes, and auxological features of the newborns. None of the pregnancies were planned despite previous counselling.

## 4 Results

*Patient 1*: She was a 43-year-old woman at the time of pregnancy affected by drug-resistant focal epilepsy due to left temporal focal cortical dysplasia. Seizures were characterized by an epigastric aura followed by impairment of awareness and automatisms. At the beginning of pregnancy, she was taking PER 8 mg/day along with zonisamide 500 mg/day. The seizure frequency was one per month, and remained stable during pregnancy. No therapeutic changes were made until delivery. Morphological ultrasounds were performed in each trimester with normal findings, and cardiac doppler ultrasound was normal in the second trimester. Pregnancy was full-term and resulted, after cesarean section, in a normal live birth without evidence of major malformations. The complete time of fetal exposure to PER was 275 days. The birth weight was 3500 g, length 49 cm, and Apgar score 9–10.

*Patient 2*: She was a 28-year-old woman at the time of pregnancy affected by drug-resistant focal epilepsy of unknown etiology. At the beginning of pregnancy, she was on treatment with PER 8 mg/day, topiramate 800 mg/day, and carbamazepine 1800 mg/day; seizures, with right sensory onset followed by impairment of awareness and automatisms, had a monthly frequency and were stable during pregnancy. Therapy remained unchanged until delivery.

There were no pathological findings in morphological ultrasounds performed every quarter of pregnancy, and cardiac doppler ultrasound was performed in the second trimester. In the 18th week, a risk of miscarriage occurred, but it spontaneously resolved. A full-term pregnancy resulted in a normal live birth after cesarean section, and no major malformations were detected. The fetus was exposed to PER for 270 days. The birth weight was 2660 g, length 47 cm, and Apgar score 9–10.

*Patient 3*: She was a 21-year-old female patient affected by drug-resistant focal epilepsy of unknown etiology at the time of pregnancy. She had been seizure-free for 18 months when taking only PER 2 mg/day, which she decided to interrupt two months before pregnancy. During the 13th week of pregnancy, the patient experienced a focal motor seizure with onset involving right head and eye deviation progressing to a bilateral tonic-clonic seizure. PER 2 mg/day was administered again and continued until birth. All morphological ultrasounds performed during pregnancy and the cardiac doppler ultrasound were normal. Pregnancy reached full term and resulted in a normal live birth via natural childbirth without evidence of major malformations. The total time of fetal exposure to PER was 185 days. The birth weight was 2980 g, length 55 cm, and Apgar score 8–9.

*Patient 4*: She was a 33-year-old pregnant patient affected by drug-resistant focal epilepsy of unknown etiology. At the beginning of pregnancy, she was taking PER 6 mg/day together with levetiracetam 3000 mg/day and oxcarbazepine 1200 mg/day. Focal onset seizures with impairment of awareness were weekly and remained stable during pregnancy. Therapy was not modified during gestation. No alterations were detected via the morphological ultrasounds performed during pregnancy, and the cardiac doppler ultrasound was normal. A live birth occurred after a full-term pregnancy with caesarean section; there was no evidence of major malformations. In total, fetal exposure to PER was 272 days. The birth weight was 2690 g, length 47 cm, and Apgar score 9–10.

The main clinical features of the patients, pregnancies, and newborns are summarized in Table 1.

**Table 1:** Main features of pregnancies and newborns.

	Patient 1	Patient 2	Patient 3	Patient 4
PER dose	8 mg/day	8 mg/day	2 mg/day	6 mg/day
Concomitant ASMs	ZNS 500 mg/day	CBZ 1800 mg/day TPM 800 mg/day	None	LEV 3000 mg/day OXC 1200 mg/day
Time of fetal exposure to PER	275 days	270 days	185 days	272 days
Pregnancy Outcome	Full term	Full term	Full term	Full term
Birth parameters	Weight: 3500 g Length: 49 cm APGAR: 9–10	Weight: 2660 g Length: 47 cm APGAR: 9–10	Weight: 2980 g Length: 55 cm APGAR: 8–9	Weight: 2690 g Length: 47 cm APGAR: 9–10
Major Malformations	None	None	None	None

ZNS = zonisamide, CBZ = carbamazepine, LEV = levetiracetam, OXC = oxcarbazepine, PER = perampanel.

## 5 Discussion

All four pregnancies demonstrated good outcomes, and none of the newborns had major malformations. The Apgar scores were normal, as were auxological parameters. Fetal follow-up during pregnancy was not pathological in any case.

In three cases, exposure to PER lasted the entire pregnancy, at a dose of 8 mg/day in two cases and 6 mg/day in the third case. PER was associated with one concomitant ASM in *patient 1* and with two concomitant ASMs in *patients 2* and *4*. *Patient 3* started pregnancy without assuming any ASM, but she started PER 2 mg/day after seizure relapse in the 13th week of gestation, and continued for the entire pregnancy; this patient should be considered apart from the others ones in relation to the beginning of the therapy after the first trimester of pregnancy, when the potential teratogenic period was exceeded.

There was only one threat of miscarriage, which occurred in *patient 2* at the 18th week and she recovered spontaneously. In the absence of seizure-related complications, caesarean section was performed in three pregnancies due to gynecological decisions.

No information about neuropsychological consequences can be provided due to the brief follow-up after delivery.

All our patients were affected by focal epilepsies, and seizure frequencies remained stable during pregnancy; in *patient 3,* no further seizures were registered after PER monotherapy was started again. Even though PER plasma concentrations were not available in our sample, the unmodified seizure frequencies during pregnancies suggest that PER plasma levels may have remained in the therapeutic range.

Considering older ASMs, clinical data in humans have always confirmed the results of preclinical studies conducted in animal models. In preclinical studies in pregnant rats and rabbits, PER doses of 1 mg/kg/day corresponded to 8 mg/day in humans [7], and no teratogenic effects emerged with doses of 1 mg, 10 mg, or 30 mg/day (Table2). To confirm preclinical data about PER, larger studies and larger registries of pregnant women exposed to the drug are needed, but reports of single cases or few pregnancies exposed to PER could be important to provide preliminary information about its safety during gestation.

**Table 2:** Perampanel during pregnancy: summary of preclinical and clinical data.

Preclinical data (animal models)					

	*ADVERSE EVENTS*				
	Dose	30–60 mg/kg/die in rats *10–60 mg/kg/die in rabbits	Post-implantation loss		
	Dose	1–3-10 mg/kg/die*	No teratogen effects Intestine diverticulum		
Clinical data					

	*AGE AT TIME OF PREGNANCY (years), n ^+^*				
	<20		7		
	20–24		13		
	25–29		17		
	30–34		13		
	35–39		16		
	≥ 40		3		
	Unknown		21		
	*NUMBER OF CONCOMITANT ASMs, n ^+^*				
	0		26		
	1		24		
	2		20		
	≥ 3		18		
	Unknown		2		
	*OUTCOMES, n ^§^*				
	Reached full term		43		
	Did not reach full term		28		
	Lost to follow-up		18		
	Ongoing pregnancy		7		
	*ADVERSE EVENTS (in pregnancies that reached full term)^#^*				
	*Newborn*	*Outcome*	*Causality*	*Perampanel dose*	*Concomitant ASMs taken*
	1	Low Apgar score	Not reported	6 mg/day	None reported
	2	Low Apgar score	Not reported	8 mg/day	None reported
	3	Neonatal aspiration (fatal)	Not related to perampanel	12 mg/day	Carbamazepine, clobazam
	4	Cystic fibrosis Congenital deafness	Not related to perampanel	Unknown	Two ASMs
	5	Poor sucking reflex-shallow breathing	Possibly related to perampanel	2 to 12 mg/day	Clonazepam

ASMs = anti-seizure medications.

*1 mg/kg/die in rats and rabbits is similar to 8 mg/die in humans.

^+^From clinical studies (n = 30) and spontaneous reports (n = 60): 90 women exposed to perampanel during pregnancies.

§ From clinical studies (n = 33) and spontaneous reports (n = 63): 96 pregnancies exposed to perampanel.

^#^From clinical studies (n = 1) and spontaneous reports (n = 4).

To date, 96 pregnancies have been reported in 90 women treated with PER (Table2), which was taken as monotherapy in 26 cases. Forty-three (44%) pregnancies reached full term, with normal live births and no major malformations described; 28 pregnancies did not reach term (induced abortion, n = 18; spontaneous or incomplete spontaneous miscarriage, n = 8; premature delivery, n = 1; stillbirth [Fallot's tetralogy], n = 1). Adverse events were reported in five newborns: low Apgar score in two cases, both on monotherapy; fatal neonatal aspiration in one case on concomitant carbamazepine and clobazam; cystic fibrosis and congenital deafness in 1 case with PER and two unspecified ASMs; poor sucking reflex and shallow breathing in one case with concomitant clonazepam [7].

Unlike previous works, which reported a low percentage (44%) of the full-term pregnancies , in our sample 3/3 pregnancies reached full term and PER showed overall a good safety profile, without side effects during pregnancies and without the occurrence of major congential malformations or adverse events in their newborns.

Compared to previous reports, our cohort details perinatal metrics and newborn vital signs and parameters of all the pregnancies observed. The small sample size, brief follow-up and unavailability of PER plasma concentrations are the main limitations of our study.

## 6 Conclusion

The increasingly use of PER in women with epilepsy requires the need for more data on safety in special conditions like pregnancy. Single case reports or data involving small case series, though promising, are not enough evidence for the lack of teratogenesis and larger study populations are needed to reach more definitive conclusions.

## Ethical publication statement

We confirm that we have read the Journal’s position on issues involved in ethical publication and affirm that this report is consistent with those guidelines.

## Declaration of Competing Interest

ALN has received speaker’s or consultancy fees from Eisai, Mylan, Sanofi, Bial, GW, Arvelle Therapeutics, Angelini Pharma and UCB Pharma. GB has received speaker’s or consultancy fees from Eisai, Angelini Pharma and UCB Pharma. The remaining authors have no conflicts of interest to declare.
